# Supplementary material for: Chemical Fingerprinting and Biological Evaluation of the Endemic Chilean Fruit Greigia sphacelata (Ruiz and Pav.) Regel (Bromeliaceae) by UHPLC-PDA-Orbitrap-Mass Spectrometry
Source: Molecules. 2020 Aug 17;25(16):3750. doi: 10.3390/molecules25163750 (PMC7464012; doi:10.3390/molecules25163750)
Supplement: Supplementary file 1 [file molecules-25-03750-s001.pdf]

Supplementary material

Chemical Fingerprinting and Biological Evaluation of the Endemic Chilean Fruit *Greigia sphacelata* (Ruiz and Pav.) Regel (Bromeliaceae) by UHPLC-PDA-Orbitrap-Mass Spectrometry

Ruth E. Barrientos [1,†], Shakeel Ahmed [1,†], Carmen Cortés [1], Carlos Fernández-Galleguillos [1], Javier Romero-Parra [2], Mario J. Simirgiotis [1,*] and Javier Echeverría [3,*]

[1] Instituto de Farmacia, Facultad de Ciencias, Universidad Austral de Chile, Valdivia 5090000, Chile; ruth.barrientos@alumnos.uach.cl (R.E.B.), shakeel.ahmed@uach.cl (S.A.), carmenc1012@gmail.com (C.C.), carlos.fernandez@uach.cl (C.F.-G.)

[2] Departamento de Química Orgánica y Fisicoquímica, Facultad de Ciencias Químicas y Farmacéuticas, Universidad de Chile, Olivos 1007, Casilla 233, Santiago, Chile, javier.romero@ciq.uchile.cl

[3] Departamento de Ciencias del Ambiente, Facultad de Química y Biología, Universidad de Santiago de Chile, Casilla 40, Correo 33, Santiago 9170002, Chile

* Correspondence: mario.simirgiotis@gmail.com or mario.simirgiotis@uach.cl (M.J.S.); javier.echeverriam@usach.cl (J.E.); Tel.: +56-63-63233257 (M.J.S.); +56-2-27181154 (J.E.)

† These authors contributed equally to this study

Received: 03 July 2020; Accepted: 14 August 2020; Published: August 2020

Keywords: *Greigia sphacelata*, endemic fruits, UHPLC-PDA-Orbitrap-MS, metabolomic analysis, antioxidant, cholinesterase inhibition

Figure S1 (a-v): Full MS spectra and structures of compounds **1, 3, 7, 9, 12, 13, 15, 25, 27, 28, 29, 35, 37, 42, 43, 44, 46, 48, 51, 53** and **55.**

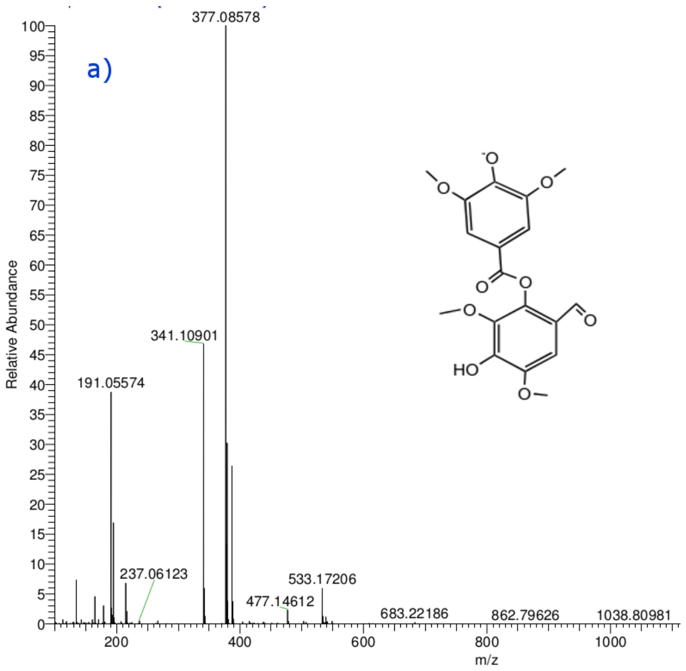

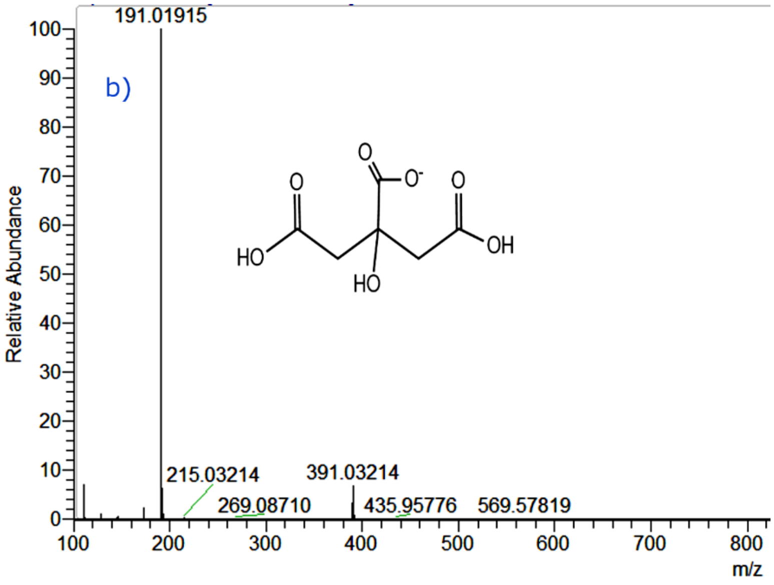

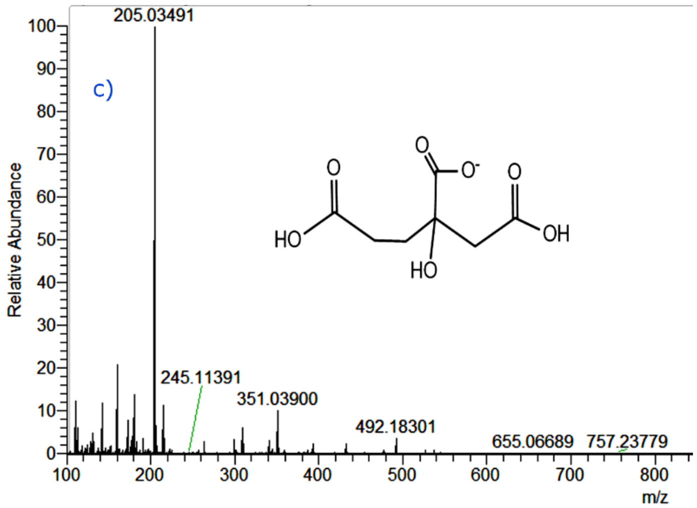

205.03491
c)
Relative Abundance
245.11391
351.03900
492.18301
655.06689
757.23779
m/z
HO
OH

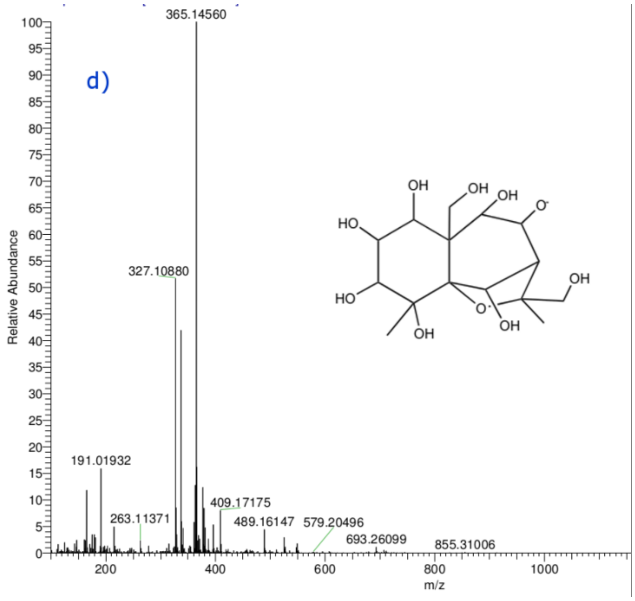

365.14560
d)
Relative Abundance
327.10880
191.01932
263.11371
409.17175
489.16147
579.20496
693.26099
855.31006
m/z
OH
HO

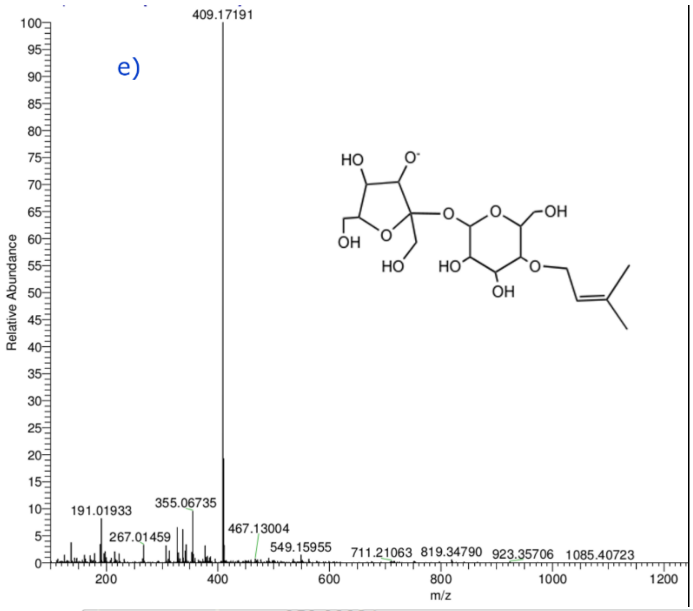
e)
409.17191
191.01933
355.06735
267.01459
467.13004
549.15955
711.21063
819.34790
923.35706
1085.40723
Relative Abundance
m/z
HO
O⁻
OH
O
OH
HO
HO
OH
O

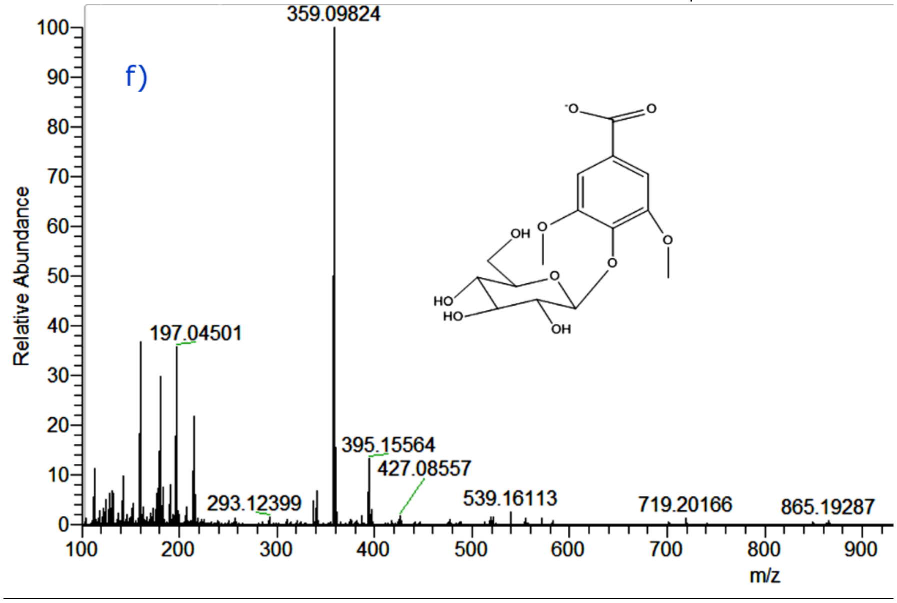
f)
359.09824
197.04501
395.15564
427.08557
293.12399
539.16113
719.20166
865.19287
Relative Abundance
m/z
⁻O
O
OH
HO
HO
OH

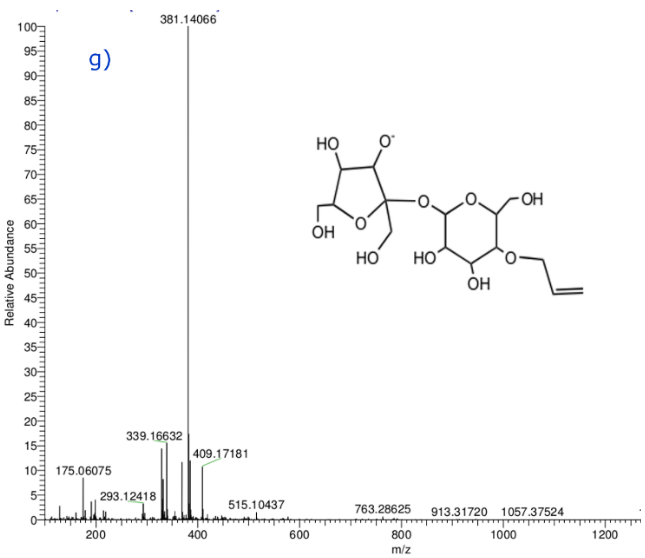

g)
381.14066
339.16632
409.17181
175.06075
293.12418
515.10437
763.28625
913.31720
1057.37524
Relative Abundance
m/z
HO
O⁻
OH
O
HO
HO
OH
O

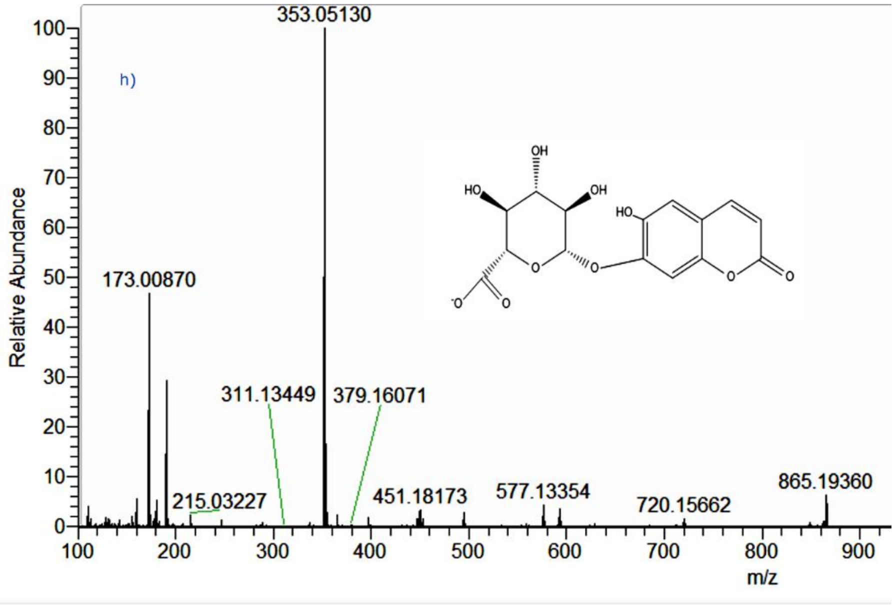

h)
353.05130
173.00870
311.13449
379.16071
215.03227
451.18173
577.13354
720.15662
865.19360
Relative Abundance
m/z
OH
HO
OH
HO
O
O
O
O

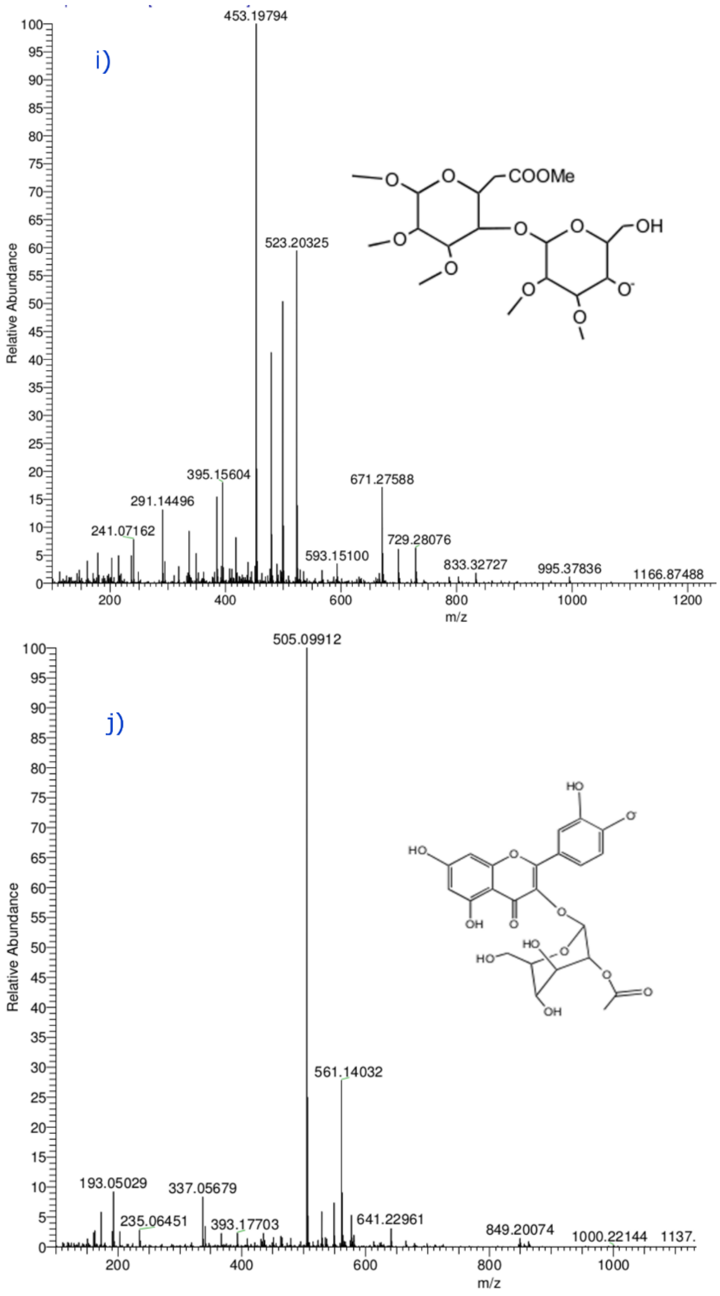
i)
453.19794
523.20325
395.15604
291.14496
241.07162
671.27588
729.28076
593.15100
833.32727
995.37836
1166.87488
Relative Abundance
m/z
COOMe
OH
j)
505.09912
561.14032
193.05029
337.05679
235.06451
393.17703
641.22961
849.20074
1000.22144
1137.
Relative Abundance
m/z
HO
OH

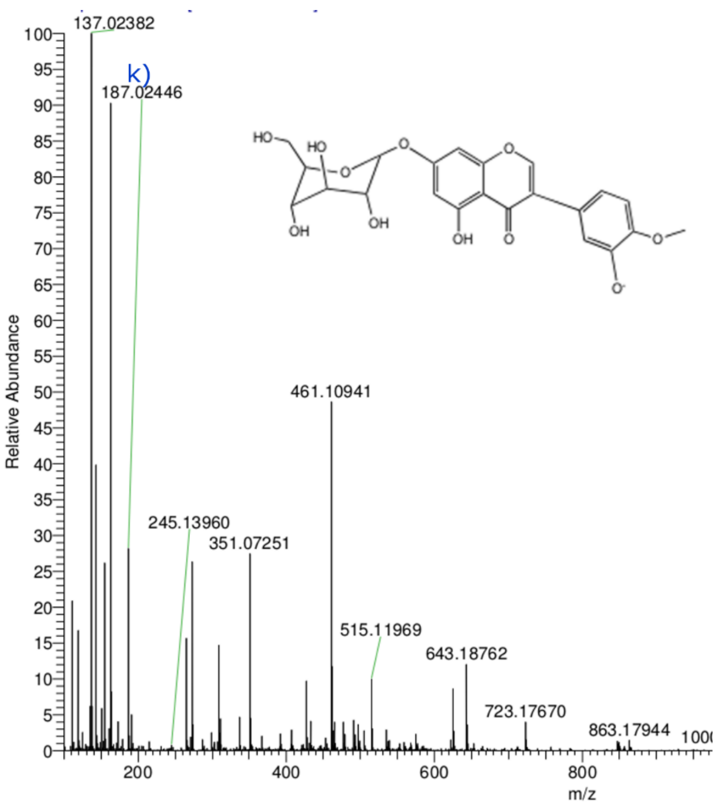
k)
137.02382
187.02446
461.10941
245.13960
351.07251
515.11969
643.18762
723.17670
863.17944
Relative Abundance
m/z

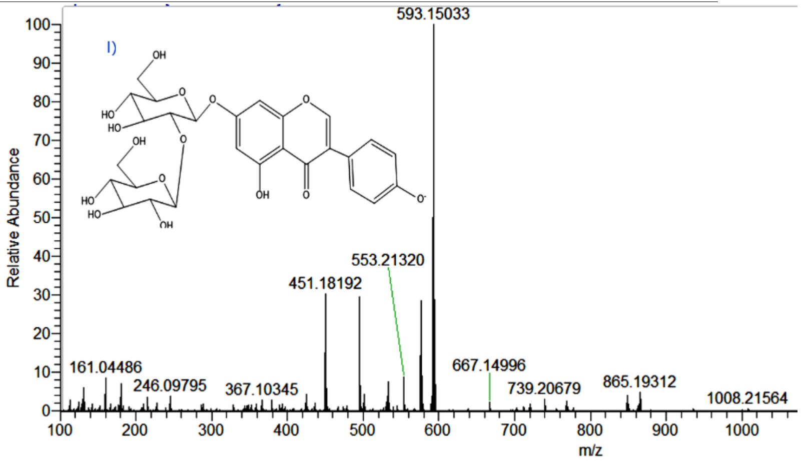
l)
593.15033
553.21320
451.18192
161.04486
246.09795
367.10345
667.14996
739.20679
865.19312
1008.21564
Relative Abundance
m/z

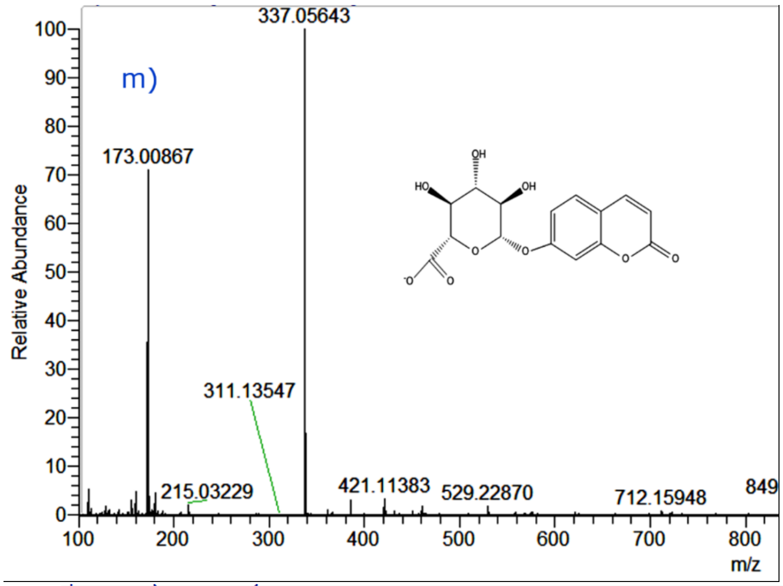
m)
337.05643
173.00867
311.13547
215.03229
421.11383
529.22870
712.15948
849
Relative Abundance
m/z
OH
HO
OH
O
O
O
O
O

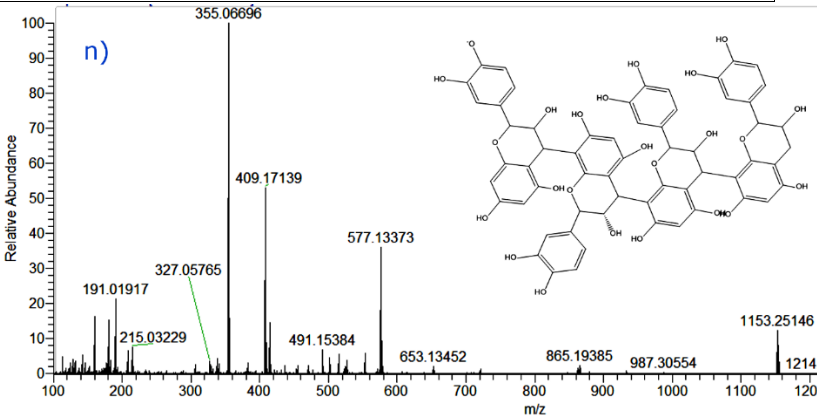
n)
355.06696
409.17139
577.13373
327.05765
191.01917
215.03229
491.15384
653.13452
865.19385
987.30554
1153.25146
1214
Relative Abundance
m/z

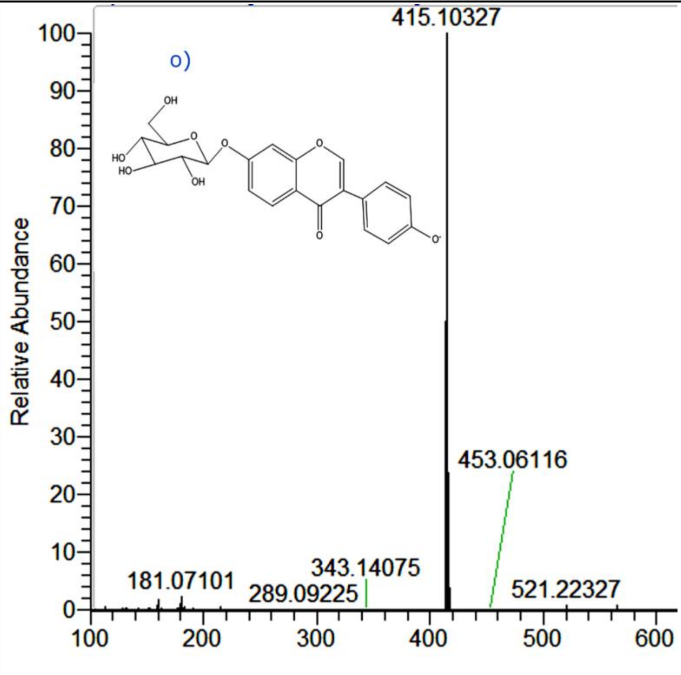
o)
415.10327
453.06116
343.14075
181.07101
289.09225
521.22327
Relative Abundance
OH
HO
HO
OH
O
O
O

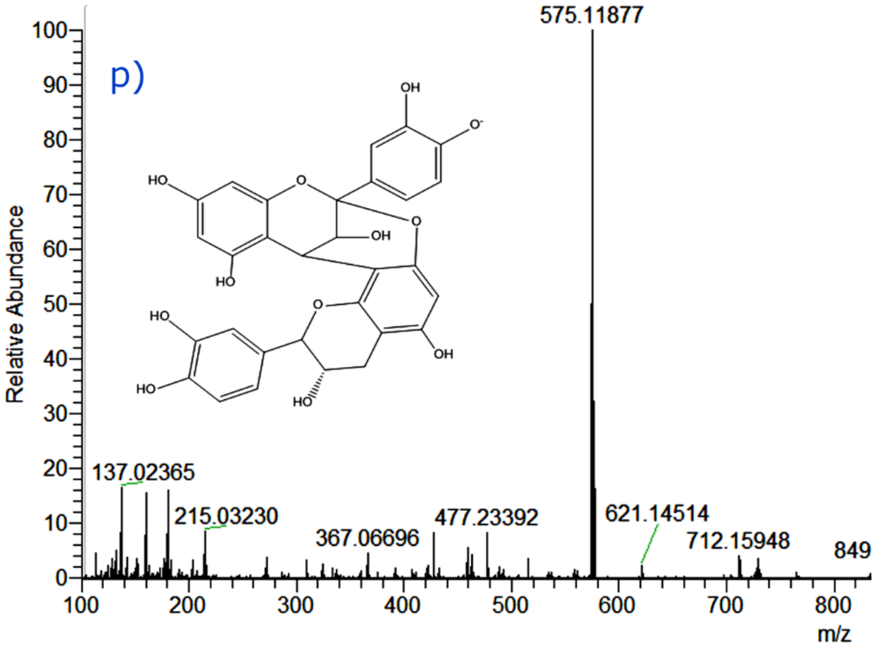

575.11877
p)
Relative Abundance
137.02365
215.03230
367.06696
477.23392
621.14514
712.15948
849
m/z

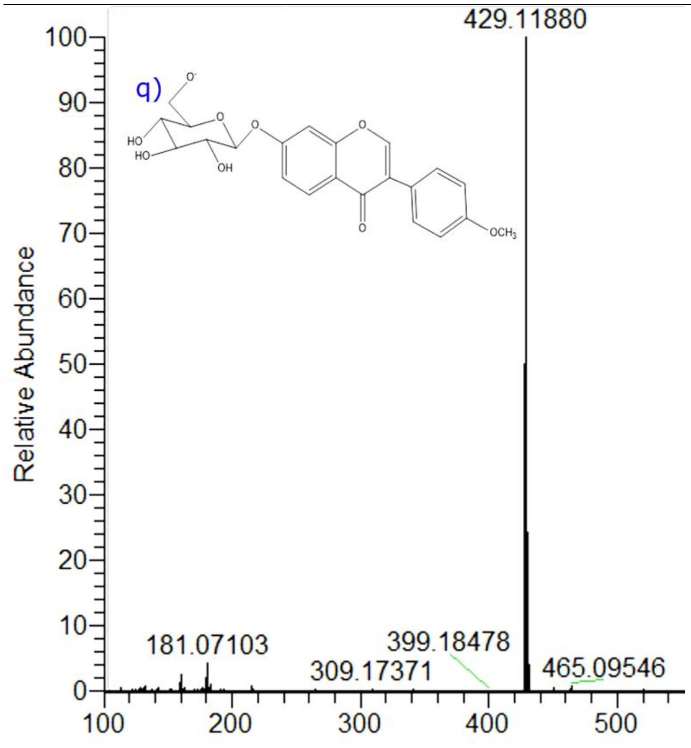

429.11880
q)
Relative Abundance
181.07103
309.17371
399.18478
465.09546

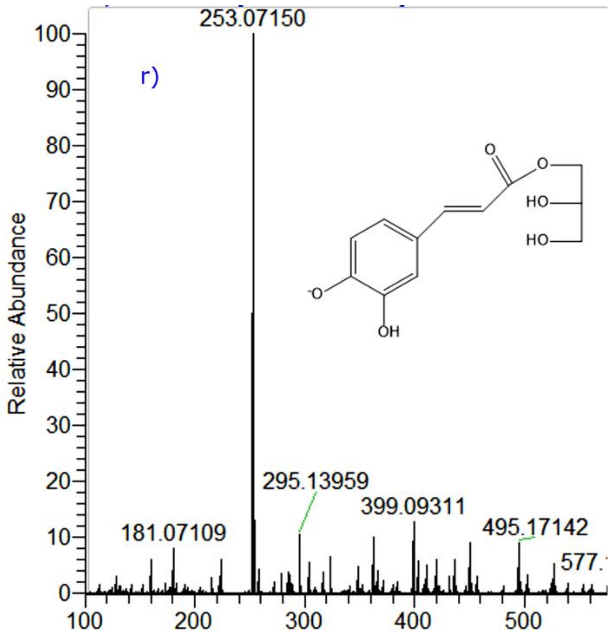
r)
Relative Abundance
253.07150
295.13959
399.09311
495.17142
181.07109
577.
HO
HO
OH

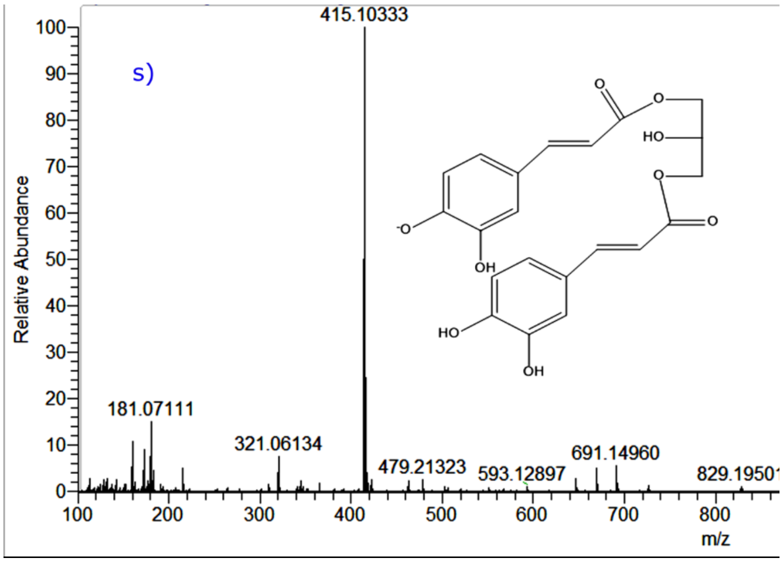
s)
Relative Abundance
415.10333
181.07111
321.06134
479.21323
593.12897
691.14960
829.1950
m/z
HO
OH
HO
OH

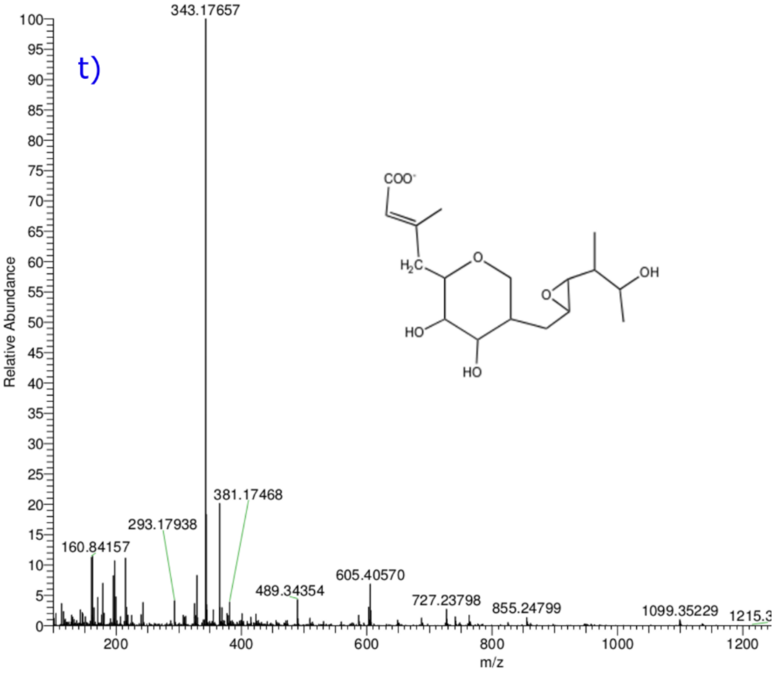
t)
343.17657
381.17468
293.17938
160.84157
489.34354
605.40570
727.23798
855.24799
1099.35229
COO-
H2C
O
HO
HO
OH
Relative Abundance
m/z

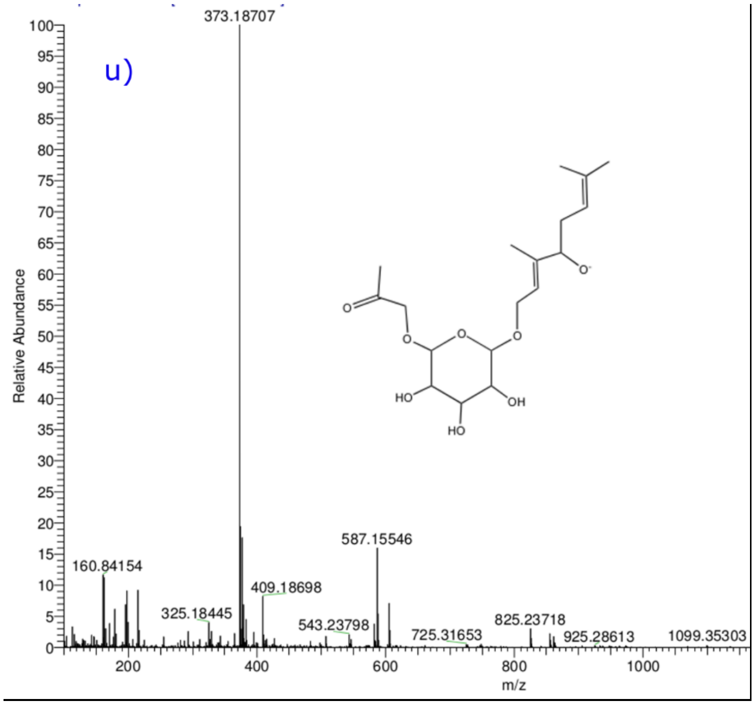
u)
373.18707
587.15546
160.84154
409.18698
325.18445
543.23798
825.23718
725.31653
925.28613
1099.35303
O-
O
HO
OH
HO
Relative Abundance
m/z

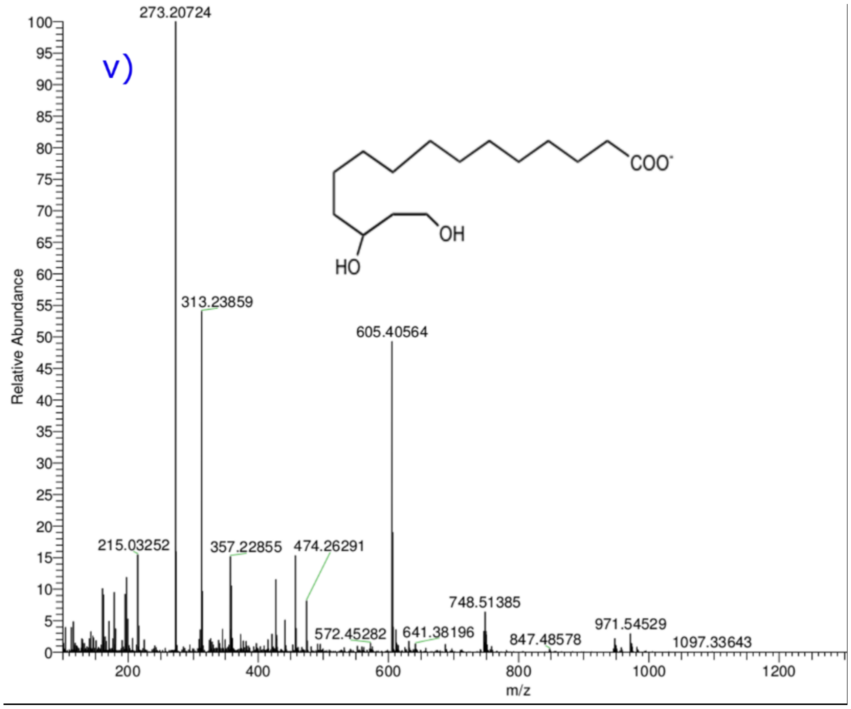
v)
273.20724
313.23859
605.40564
215.03252
357.22855
474.26291
748.51385
572.45282
641.38196
847.48578
971.54529
1097.33643
COO-
OH
HO
Relative Abundance
m/z

Docking analyses of *Greigia sphacelata* main compounds

Material and Methods

Docking simulations were carried for those compounds (**Figure S1**) that turned out to be the most abundant species according to the UHPLC Chromatogram (**Figure 2**) obtained from the pulp and seeds of the *G. sphacelata*'s fruit. The geometries and partial charges of quercetin-3-*O*-glucoside acetate (peak **28**), lupinisoflavone A (peak **32**), genistein-7-*O*-di-glucoside (peak **35**), ononin (formononetin 7-*O*-glucoside) (peak **46**), genistein-7-*O*-glucoside (peak **38**), aesculetin-*O*-glucuronide (peak **25**), dihydroxy-octadecaenoic acid (peak **62**), and hydroxy-pentadecanoic acid (peak **69**) were fully optimised using the DFT method with the standard basis set PBE0/ 6-311+g*[1, 2]. All calculations were performed in Gaussian 09W software[3]. Crystallographic enzyme structures of *Torpedo californica* acetylcholinesterase (*Tc*AChE; PDBID: 1DX6 code[4]) and human butyrylcholinesterase (*h*BuChE; PDBID: 4BDS code[5]) were downloaded from the Protein Data Bank RCSB PDB[6]. Water molecules and ligands of the crystallographic protein active sites were removed. All polar hydrogen atoms of both enzymes were added and proteins were treated as rigid bodies. Grid maps were calculated using the Autogrid option and were centred on the putative catalytic site of each enzyme considering their known catalytic residues: Ser200, Glu327 and His440 for *Tc*AChE [7, 8] and Ser198, Glu325 and His438 for *h*BuChE [9, 10] respectively. Ser200 of *Tc*AChE and Ser198 of *h*BuChE were designated as the centre of the grids for the catalytic site of each enzymes. The volumes chosen for the grid maps for both catalytic sites were made up of 60 × 60 × 60 points in the x, y, z directions of 6.634, 63.588, 60,192 for *Tc*AChE and 138.839, 120.098 and 41.943 for *h*BuChE, respectively. A grid-point spacing of 0.375 Å was established. Docked compound complexes were built using the Lamarckian Genetic Algorithm[11] which involved 100 runs. The lowest docked-energy binding cluster positions were chosen to be analyzed according to the potential intermolecular interactions between compounds and the enzymes, as well as to obtain the binding mode and docking descriptors. The different complexes were visualised in a Visual Molecular Dynamics program (VMD) and Pymol [12].

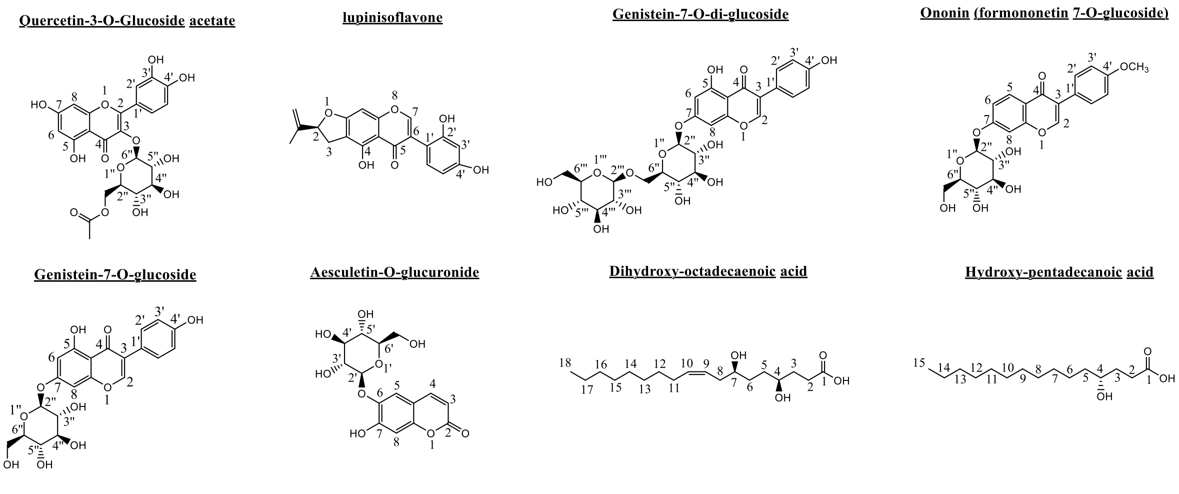

Figure S2. Components of *G. sphacelata* fruits used in docking studies.

Results and Discussion

All Compounds that turned out to be the most abundant species according to the UHPLC Chromatogram (**Figure 2**) obtained from the pulp and seeds of *G. sphacelata*, as well as the known cholinesterase inhibitor galantamine, were subjected to docking assays into the *Tc*AChE catalytic site and *h*BuChE catalytic site, in order to rationalize their pharmacological results analyzing their protein molecular interactions in the light of their experimental inhibition activities showed in **Table 2**. The best docking binding energies expressed in kcal/mol of each compound are shown in **Table S1**.

Table S1. Binding energies obtained from docking experiments of most abundant compounds in *G. sphacelata*'s fruit and the known cholinesterase inhibitor galantamine over acetylcholinesterase (*Tc*AChE) and butyrylcholinesterase (*h*BuChE).

**Compound**	**Binding energy (kcal/mol) Acetylcholinesterase (*Tc*AChE)**	**Binding energy (kcal/mol) Butyrylcholinesterase (*h*BuChE)**
**Quercetin-3-*O*-glucoside-acetate**	-9.46	-8.31
**Lupinisoflavone**	-9.36	-7.99
**Genistein-7-*O*-di-glucoside**	-9.18	-6.89
**Ononin (formononetin 7-*O*-glucoside)**	-7.45	-6.44
**Genistein-7-*O*-glucoside**	-7.24	-5.86
**Aesculetin-7-*O*-glucuronide**	-6.67	-6.85
**Dihydroxy-octadecaenoic acid**	-4.71	-5.76
**Hydroxy-pentadecanoic acid**	-4.81	-4.87
**Galantamine**	-11.81	-9.5

Acetylcholinesterase (TcAChE) docking results

Table S1 showed that the flavonoid quercetin-3-*O*-glucoside-acetate, and the isoflavones Lupinisoflavone and Genistein-7-*O*-di-glucoside displayed the best binding energies of -9.46, -9.36 and -9.18 kcal/mol, respectively. These results suggest that *G. sphacelata* pulp or seed extracts inhibitory activity over acetylcholinesterase are mainly due the compounds mentioned above, especially the flavonoid quercetin-3-*O*-glucoside-acetate.

Quercetin-3-*O*-glucoside-acetate perform five different hydrogen bond interactions; two of them are carried out between the hydrogen atoms of the hydroxyl groups (-OH) at position 3′- and 4′- of the phenyl ring of the 4*H*-chromen-4-one framework and the amino acid Glu199. The 4′- hydroxyl group (-OH) also shows a hydrogen bond interaction with Tyr130 through its oxygen atom (**Figure S3**). Moreover, since the 4*H*-chromen-4-one moiety of quercetin-3-*O*-glucoside-acetate possesses two more hydroxyl groups at positions 5- and 7-, each one of them are in charge to perform another hydrogen bond interaction stabilizing the protein–inhibitor complex. The good binding energy value shown by quercetin-3-*O*-glucoside-acetate may be supported by these hydrogen bond interactions profile mentioned above.

Lupinisoflavone exhibit a binding energy value of -9.36 kcal/mol and shows two hydrogen bond interactions through the hydroxyl group (-OH) of phenyl the moiety at position 6- of the 2,3-dihydro-5*H*-furo-chromen-5-one and the amino acids Glu199 and Tyr130. Isoflavone also perform two extra π-π interactions among the residues of Phe288 and Phe 331 with the aromatic rings of benzene in the dihydro-5*H*-furo-chromen-5-one core and the phenyl moiety at posotion 6-. Especially, the first π-π mentioned interaction is probably favoured because of its quite flat chemical structure due its three fused rings.

Even though quercetin-3-*O*-glucoside-acetate and Lupinisoflavone structures are arranged in different manners into the *Tc*AChE catalytic site, these derivatives were the only compounds of all those shown **Table 2** that presented these hydrogen bond interactions with the same Glu199 and

Tyr130 amino acids at the same time, suggesting that these two residues could play a key role in the *Tc*AChE inhibition when an inhibitor interacts with them.

Genistein-7-*O*-di-glucoside has a binding energy value of -9.18 kcal/mol and shows four hydrogen bond interactions with Asp72, Glu199, Ser200 and Ser286, as well as a π-π interaction between the benzene ring of the 4*H*-chromen-4-one scaffold and Tyr334 (**Figure S3**). Thus, this derivative into the extract represent a good candidate to behave as a *Tc*AChE inhibitor compared to ononin (formononetin 7-*O*-glucoside), genistein-7-*O*-glucoside, aesculetin-7-*O*-glucuronide, dihydroxy-octadecaenoic acid and hydroxy-pentadecanoic acid.

Ononin (formononetin 7-*O*-glucoside) and Genistein-7-O-glucoside possesses resembling chemical structures; hence are settled in similar modes into the *Tc*AChE catalytic site. The latter could be the reason why these derivatives share some interactions with the same amino acids, such as Tyr130 and Phe288, as well as similar binding energy values (-7.45 and -7.24 kcal/mol respectively, see **Table 3**).

The coumarin aesculetin-7-O-glucuronide exhibit only two hydrogen bond interactions with Glu199 through the two hydroxyl groups (-OH) of the glucoronide moiety at positions 3'- and 5' and a binding energy of -6.67 kcal/mol. Therefore, this derivative, as well as the dihydroxy-octadecaenoic acid and the hydroxy-pentadecanoic acid which carry out only few interactions with the catalytic site of the enzyme, presents low binding energies and have no possibilities to perform other sorts of interactions like π-π or T-shaped. Thus, these compounds would not contribute to the enzyme inhibition in a significant manner, even if they are in high proportion into the extracts (**Figure S3**).

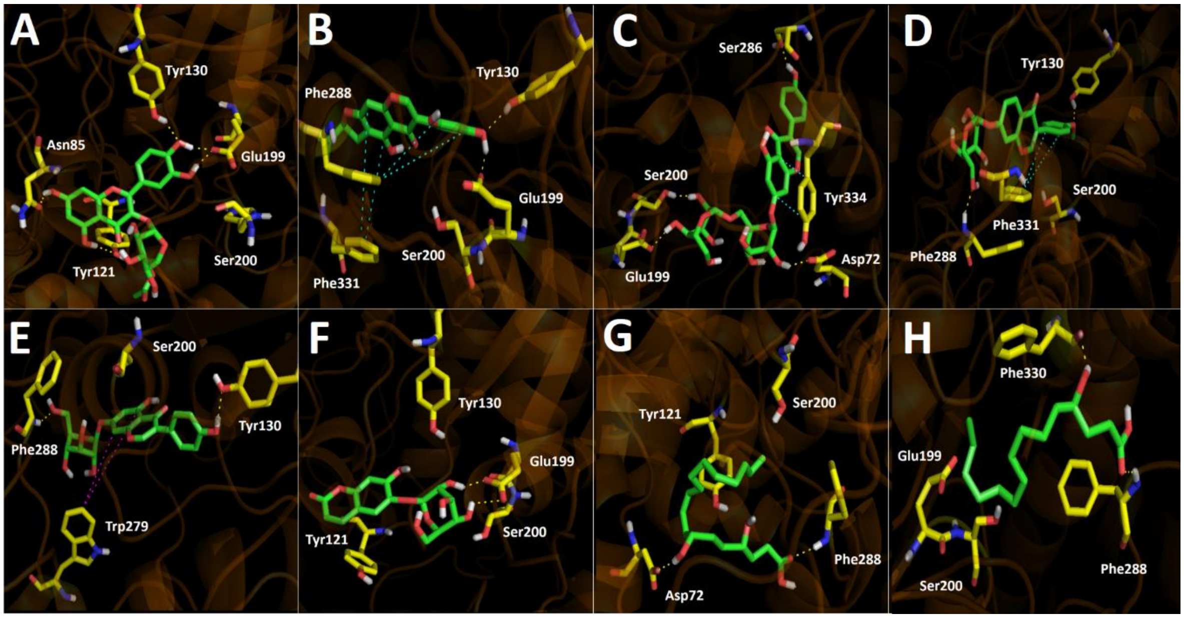

Figure S3. Predicted binding mode and predicted intermolecular interactions of all most abundant compounds in *G. sphacelata* pulp and seeds extracts and the residues of *Torpedo californica* acetylcholinesterase (*Tc*AChE) catalytic site. Yellow dotted lines indicate hydrogen bond interactions, cyan dotted lines represent π-π interactions. **A.** Quercetin-3-*O*-glucoside-acetate (flavonoid) into the catalytic site; **B.** Lupinisoflavone (isoflavone) into the catalytic site; **C.** Genistein-7-*O*-di-glucoside (isoflavone) into the catalytic site; **D.** Ononin (formononetin 7-*O*-glucoside) (isoflavone) into the catalytic site; **E.** Genistein-7-*O*-glucoside (isoflavone) into the catalytic site **F.** Aesculetin-7-*O*-glucuronide (coumarin) into the catalytic site **G.** Dihydroxy-octadecaenoic acid (fatty acid) into the catalytic site; **H.** Hydroxy-pentadecanoic acid (fatty acid) into the catalytic site.

Pulp and seeds extract presented considerably abilities to exert an inhibitory potency over the *Tc*AChE enzyme (IC_{50}= 4.94 ± 0.075 for pulp extract and IC_{50}= 4.98 ± 0.042 for seeds extract) considering the known cholinesterase inhibitor galantamine (see table 3). In this sense, **Figure S3** shows the hydrogen bond interactions in a two dimensional diagram of each main and most abundant compounds determined from both extracts into the *Tc*AChE catalytic site in order to summarize the information.

Butyrylcholinesterase (hBuChE) docking results

All binding energies obtained from docking assays over butyrylcholinesterase (*h*BuChE) of the most abundant compounds in the pulp and the seeds extracts showed to be poorer compared to those in *Tc*AChE. These results are consistent with the less inhibitory activity of the extracts over this enzyme shown in table 2 (IC_{50}= 73.86 ± 0.086 for pulp extract and IC_{50}= 78.57 ± 0.064 for seeds extract).

Just like in *Tc*AChE, the flavonoid quercetin-3-*O*-glucoside-acetate exhibited the best binding energy profile, suggesting that this derivative could be the main responsible for the inhibitory activity over the *h*BuChE.

Quercetin-3-*O*-glucoside-acetate binding descriptors over *h*BuChE had certain differences compared to those shown by *Tc*AChE. Quercetin-3-*O*-glucoside-acetate into the *h*BuChE catalytic site perform only three hydrogen bond interactions. Two of them are carried out between the hydroxyl group (-OH) at position 4' in the phenyl moiety of the 4*H*-chromen-4-one framework and the amino acids Glu197 and Tyr128. Moreover, this flavonoid performs a π-π interaction between the catechol ring and the residue of Trp231, and two T-shaped interactions between the catechol ring and the benzene scaffold of the 4*H*-chromen-4-one core as well (**Figure S4**). Nevertheless, the lack of hydrogen bond interactions compared to those carried out by quercetin-3-*O*-glucoside-acetate into the *Tc*AChE catalytic site could explain the less binding energy of the *h*BuChE–quercetin-3-*O*-glucoside-acetate complex.

Lupinisoflavone's *h*BuChE binding energy, as well as that obtained for *Tc*AChE docking assays, presented the second-best profile, hence it could contribute to the inhibitory activity of the extracts over the enzyme. Nonetheless, also this derivative presented less binding energy relating to the obtained over *Tc*AChE docking results, as was aforementioned. Lupinisoflavone perform a hydrogen bond interaction between the oxygen atom of the dihydrofuran ring at position 1- and the residue of His438. Also present a second hydrogen bond interaction through the oxygen atom of the carbonyl of Trp82 and the hydroxyl group (-OH) at position 2′ of the phenyl moiety. Both distant interactions can be done owing its flat chemical structure.

Isoflavonoids genistein-7-*O*-di-glucoside, ononin (formononetin 7-*O*-glucoside) and genistein-7-*O*-glucoside due their similar chemical structures are overlapped among them into the *h*BuChE catalytic site, and therefore exhibit related binding modes and similar binding energies as can be seen in table 3. The three isoflavonoids mentioned above perform the same hydrogen bond interaction with His438 through one of the hydroxyl groups (-OH) their glycoside moieties. Likewise, genistein-7-*O*-di-glucoside and ononin (formononetin 7-*O*-glucoside) carry out another hydrogen bond interaction through the 3′′-OH of their glycosides scaffolds with Trp82, but genistein-7-*O*-glucoside perform the same interaction through its 3′′-OH with the nearby amino acid Trp430. Genistein-7-*O*-di-glucoside expose a substantial difference compared to the other two isoflavonoids, carrying out an extra hydrogen bond interaction between the 4' phenolic hydroxyl (-OH) and Asn68, what the

other derivatives do not perform. This latter feature could explain the best binding energy exhibited by genistein-7-*O*-di-glucoside (-6.89 kcal/mol).

The coumarin aesculetin-7-*O*-glucuronide, as well as the fatty acids dihydroxy-octadecaenoic acid and hydroxy-pentadecanoic acid, like in *Tc*AChE docking assays, showed the worse binding energies, and therefore they are probably not significant contributors for the *h*BuChE inhibition. Aesculetin-7-*O*-glucuronide shows two hydrogen bond interactions with Glu197 through two hydroxyl groups (-OH) of its glycoside core, and the fatty acids also carry out poor interactions being only two hydrogen bond interactions in charge of stabilizing the protein–compound complex in the dihydroxy-octadecaenoic acid (interactions with Gly117 and Glu197) and only one hydrogen bond interaction between the hydroxyl (-OH) at position 4- of the aliphatic chain and Gly117. All binding mode positions and descriptors of each abundant compounds obtained from *G. sphacelata* pulp and seeds extracts over the *h*BuChE are shown in **Figure S4**.

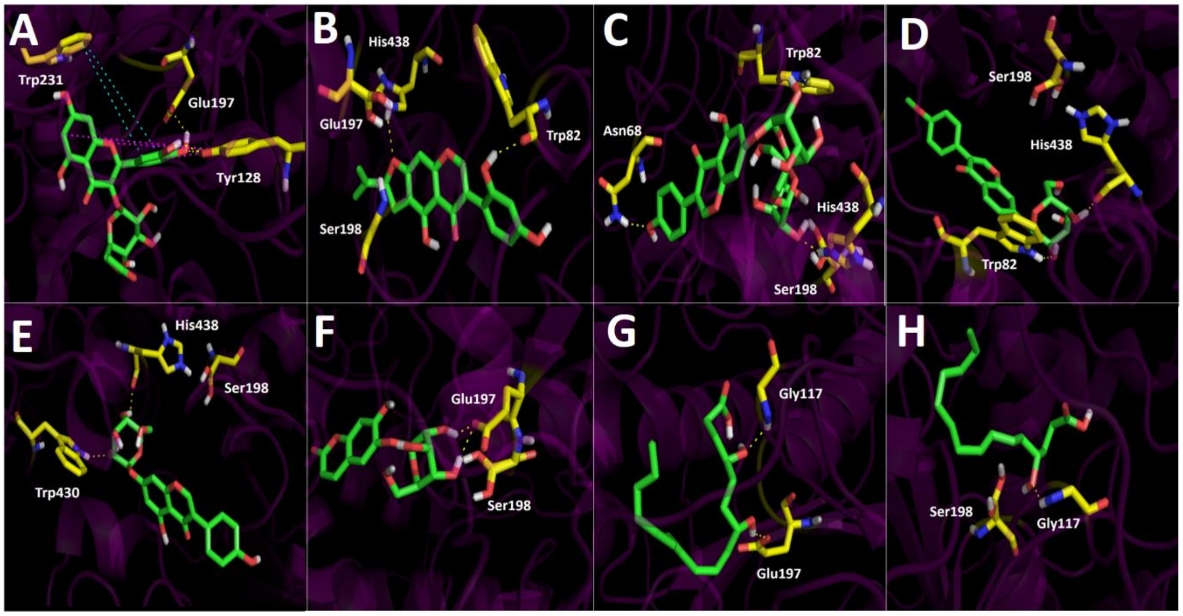

Figure S4. Predicted binding mode and predicted intermolecular interactions of all most abundant compounds in *G. sphacelata* pulp and seeds extracts and the residues of human butyrylcholinesterase (*h*BuChE) catalytic site. Yellow dotted lines indicate hydrogen bond interactions, cyan dotted lines represent π-π interactions and magenta dotted lines indicates T-Shaped interactions. **A.** Quercetin-3-*O*-glucoside-acetate (flavonoid) into the catalytic site; **B.** Lupinisoflavone (isoflavone) into the catalytic site; **C.** Genistein-7-*O*-di-glucoside (isoflavone) into the catalytic site; **D.** Ononin (formononetin 7-*O*-glucoside) (isoflavone) into the catalytic site; **E.** Genistein-7-*O*-glucoside (isoflavone) into the catalytic site **F.** Aesculetin-7-*O*-glucuronide (coumarin) into the catalytic site **G.** Dihydroxy-octadecaenoic acid (fatty acid) into the catalytic site; **H.** Hydroxy-pentadecanoic acid (fatty acid) into the catalytic site.

References

1. Adamo, C. and V. Barone, *Toward reliable density functional methods without adjustable parameters: The PBE0 model.* The Journal of chemical physics, 1999. **110**(13): p. 6158-6170.
2. Petersson, a., et al., *A complete basis set model chemistry. I. The total energies of closed-shell atoms and hydrides of the first-row elements.* The Journal of chemical physics, 1988. **89**(4): p. 2193-2218.
3. Frisch, A., *Gaussian 09 User's Reference*. 2009: Gaussian, Incorporated.
4. Greenblatt, H., et al., *Structure of acetylcholinesterase complexed with (−)-galanthamine at 2.3 Å resolution.* Febs Letters, 1999. **463**(3): p. 321-326.
5. Nachon, F., et al., *Crystal structures of human cholinesterases in complex with huprine W and tacrine: elements of specificity for anti-Alzheimer's drugs targeting acetyl-and butyryl-cholinesterase.* Biochemical Journal, 2013. **453**(3): p. 393-399.
6. Berman, H., et al., *RCSB Protein Data Bank: Structural biology views for basic and applied research.* Nucleic Acids Res, 2000. **28**: p. 235-242.
7. Silman, I., et al., *Three-dimensional structures of acetylcholinesterase and of its complexes with anticholinesterase agents*. 1994, Portland Press Limited.
8. Sussman, J.L., et al., *Atomic structure of acetylcholinesterase from Torpedo californica: a prototypic acetylcholine-binding protein.* Science, 1991. **253**(5022): p. 872-879.
9. Nicolet, Y., et al., *Crystal structure of human butyrylcholinesterase and of its complexes with substrate and products.* Journal of Biological Chemistry, 2003. **278**(42): p. 41141-41147.
10. Tallini, L., et al., *Cholinesterase Inhibition Activity, Alkaloid Profiling and Molecular Docking of Chilean Rhodophiala (Amaryllidaceae).* Molecules, 2018. **23**(7): p. 1532.
11. Thomsen, R. and M.H. Christensen, *MolDock: a new technique for high-accuracy molecular docking.* Journal of medicinal chemistry, 2006. **49**(11): p. 3315-3321.
12. DeLano, W.L., *The PyMOL molecular graphics system.* http://www. pymol. org, 2002.
